# Advanced Research on Structure–Function Relationships of Membrane Proteins

**DOI:** 10.3390/membranes12070672

**Published:** 2022-06-29

**Authors:** Akira Naito, Izuru Kawamura

**Affiliations:** Graduate School of Engineering, Yokohama National University, 79-5 Tokiwadai, Hodogaya-ku, Yokohama 240-8501, Japan

Membrane proteins embedded in biological membranes account for 30% of the proteins encoded in the human genome and play an essential role in maintaining the homeostasis of cells by functioning as transporters, for signal transaction and energy conversion, amongst other functions. Thus, knowledge of the atomic resolution structures of membrane proteins is extremely crucial to understanding their functions. However, it is difficult to determine the structure of membrane proteins at the atomic resolution, compared to soluble proteins, because of the degree of their crystallization.

Recent developments in the methodology of structure determination are providing high-resolution structures of membrane proteins, and several high-resolution structures of membrane proteins have already been reported (~4% of PDB). Solid-state NMR spectroscopy does not require crystallization and is not restricted by upper limits to the molecular weight and structure of the heptahelical membrane protein being determined [1]. Solution NMR spectroscopy has recently been used to determine the structure of membrane proteins using detergent micelles or nanodiscs as membrane mimic systems [2]. For X-ray crystallography, it is becoming possible to determine the structures of membrane proteins by using membrane or membrane mimic systems to form the crystals [3]. The recently developed time-resolved methods allow for a determination of the structures of the short-lived intermediates of membrane proteins [4]. Attention is now being focused on cryo-electron microscopy (cryo-EM) as a powerful method for determining the structure of membrane proteins using two-dimensional crystals [5]. The determination of membrane protein structure based on single-molecular cryo-EM observation has also recently become possible [6].

This Special Issue focuses on advanced studies of structure–function relationships using advanced methods to determine the high-resolution structures of membrane proteins, including developments in methodology. Within the scope of this Special Issue are not only determinations of complete structures but also of the functions related to the local structural changes and the dynamic properties of membrane proteins as summarized in Figure 1.

The article in this Special Issue by Florian Cymer et al. [7] reports that the single transmembrane helix of human carbonic anhydrases XII of membrane proteins contains a large number of amino acids with small side chains and suggests the critical roles of these small amino acids in the dimerization of the transmembrane domain. Using the GALLEX assay, the authors show that the transmembrane domain forms a strong transmembrane helix oligomer embedded in a biological membrane. The authors also showed that single or multiple mutations of small residues to isoleucine consistently increased the interaction propensities. A reduction of the helix flexibility and of the protein–lipid contacts may increase stability of helix–helix interactions within the membrane.

In the review by Koh Takeuchi et al. [8], a solution NMR has been highlighted to reveal the structural basis of the function of multi-spanning membrane proteins, such as ion channels, GPCRs, and transporters. Furthermore, membrane proteins are under conformational equilibrium between different conformational states that are associated with distinctive functional states. The relative population of the different conformational states can quantitatively reveal the functionality of the membrane protein under certain conditions. The exchange rates between the distinct conformational states described by solution NMR can ultimately define the time scale of the cell response as investigated for ion channels and GPCRs. Since solution NMR allows for the detecting and quantifying of proton dynamics at various timescales and at a semi-atomic resolution, the method is suitable for understanding the functions of the multi-spanning helical membrane proteins.

The article by Cuauhtemoc U. Gonzalez et al. [9] explores the research on the structural arrangement produced by concanavalin A (Con-A) binding to homomeric GluK2 receptors. Con-A stabilizes the active open-channel state of the kainate receptor and reduces the extent of desensitization. In this study, the authors used single-molecule fluorescence resonance energy transfer (smFRET) methods to investigate the conformational changes underlying kainate receptor modulation by Con-A. These studies showed that Con-A binding to Gluk2 receptor is located close to the subunit at the dimer–dimer interface at the amino-terminal domain and between the subunits at the agonist-binding domain. Based on these results, the authors conclude that Con-A modulation of kainate receptor function is mediated by a shift in the conformation of the kainate receptor toward a tightly packed extracellular domain.

The excellent review by Saman Majeed et al. [10] focuses on the most widely used membrane mimetics in structural and functional studies of integral membrane proteins (IMPs) at the molecular level. These membrane mimetics are detergents, liposomes, bicelles, nanodiscs/Lipodisqs, amphipols, and lipidic cubic phases. The authors also discuss the protocols for IMP reconstitution in membrane mimetics as well as the applicability of these membrane mimetic-IMP complexes in studies using a variety of biochemical, biophysical, structural, and structural-biological techniques. The diversity of these systems has grown significantly, and the widely ranging lipid membrane-mimetic platforms ca now provide high solubility, stability, more or less lipid-bilayer environments, and other specific properties that are utilized in studies using solution NMR, X-ray crystallography, cryo-EM, ESR, fluorescence spectroscopy assays, ligand binding, translocation assays, etc.

Chun-Hao Liu et al. [11] examine the effect of cholesterol on the interaction of viral protein R (Vpr) and lipid membranes. Using a calcein release assay, the authors found that the membrane permeability induced by the membrane binding of Vpr was significantly reduced in the presence of cholesterol in the membrane. Using solid-state NMR spectroscopy, Vpr was shown to experience inhomogeneous chemical environments as shown in the broad ^13^CO NMR signal of Cys-76 residue ranging from 165-178 ppm, which can be attributed to the existence of multiple Vpr-membrane environments. The authors revealed that the presence of cholesterol in the membrane will alter the distribution of Vpr in the multiple membrane environments, which may explain the change of the Vpr induced membrane permeability in the presence of cholesterol.

In the review by Shinya Hanashima et al. [12], molecular level structural analyses of the interaction between membrane proteins and lipids or detergents that constitute biological or artificial model membranes are shown to be important for understanding the functions and physicochemical properties of membrane proteins and biomembranes. A determination of membrane protein structures at the molecular level is much more difficult when compared with that of soluble proteins, but the new technical development has accelerated the elucidation of the structure–function relationship of membrane proteins. The authors summarize the development of heavy atom derivative detergents and lipids that can be used for a molecular level structural analysis of membrane proteins, including their application for short lived intermediates with X-ray free-electron laser crystallography.

The article by Munehiro Kumashiro et al. [13] reports that the formation of β-strand oligomers of antimicrobial peptide magainin 2 (M2) contributed to the disruption of a phospholipid membrane. The authors measured the synchrotron-radiation circular dichroism and linear dichroism (LD) spectra of M2 in lipid membranes to characterize the conformation and orientation of M2 on the membrane. A singular value decomposition analysis supported the presence of the intermediate state, and a global fitting analysis revealed that M2 monomers with an α-helix structure assembled to form M2 oligomers with a β-strand-rich structure in the intermediate state, which was observed using LD spectra. Fluorescence spectroscopy showed that the formation of β-strand oligomers destabilized the membrane packing structure and induced the leakage of calcein. These results suggest that the formation of β-strand oligomers of M2 plays a crucial role in the disruption of the cell membrane.

The review of Nadezhda Barvitenko et al. [14] indicates that endothelial mechanosensors are the key upstream signaling proteins in shear stress (SS)-induced pro- and anti-inflammatory responses. As transmembrane proteins, mechanosensors not only experience fluid SS but also become regulated by the biomechanical properties of the lipid bilayer and the cytoskeleton. The authors highlight the apparent effects of pro-inflammatory factors (hypoxia, oxidative stress, hypercholesterolemia, and cytokines) on the biomechanics of the lipid bilayer and the cytoskeleton. An analysis of the data reveals that the formation of a vicious circle may occur, in which pro-inflammatory cytokines enhance and attenuate SS-induced pro-inflammatory and anti-inflammatory signaling, respectively.

Yee-Shan Ku et al. [15] investigate the phenomenon whereby multidrug and toxic compound extraction (MATE) transporters in eukaryotes have been characterized to be antiporters that mediate the transport of substrates in exchange for protons. By performing phylogenetic analyses and a protein sequence alignment of 14 representative plant species, the authors identified a distinctive N-terminal poly-glutamate motif among a cluster of MATE proteins in soybeans. Amongst them, GmMATE4 has the most consecutive glutamate residues at the N-terminus. A subcellular localization study showed that GmMATE4 was localized at the vacuolar membrane-like structure. Using yeast as the model, the authors showed that GmMATE4 mediated the transport of daidzein, genistein, glycitein, and glycitin. In summary, the authors demonstrated that GmMATE4 acts as an isoflavone transporter and revealed the functional significance of the N-terminal poly-glutamate motif of GmMATE4 in regulating the isoflavone transport activity.

In the article of Arisu Shigeta et al. [16], a novel technique for in situ photoirradiation of ^13^C solid-state NMR was developed and used to observe a variety of photo-intermediates and photoreaction pathways in [20-^13^C]retinal-WT-BR and its mutant [20-^13^C, 14-^13^C]retinal-D96N-BR. In WT-BR, the CS state converted to the CS* intermediate under green light irradiation at −20 °C and consequently converted to the AT state in the dark. The AT state converted to the N intermediate under green light irradiation. In D96N-BR, the CS state was converted to the CS* intermediate at −30 °C and consequently converted to the AT state. Simultaneously, the AT state converted to the M and L intermediates under green light irradiation at −30 °C and subsequently converted to the AT state in the dark. The authors demonstrated that short-lived photo-intermediates could be observed in a stationary state using in situ photoirradiation solid-state NMR spectroscopy for WT-BR and D96N-BR, enabling insights into the light-driven proton pump activity of BR.

Matthew J. Laurence et al. [17] reviewed current nanodisc techniques and the fundamentals of fluorescence correlation spectroscopy (FCS). The development of nanodisc techniques, such as nanolipoprotein particles (NLPs) and styrene maleic acid polymers (SMALPs), allow membrane proteins to be expressed and isolated in solution as part of lipid bilayer rafts with defined, consistent nanometer sizes and compositions, thus enabling solution–based measurements. FCS is a relatively simple yet powerful optical microscopy-based technique that provides quantitative biophysical information, such as diffusion kinetics and concentrations, about individual or interacting membrane proteins in solution.

In conclusion, six research articles and five review articles have been included in this Special Issue. Structural, dynamics, and interaction studies on membrane proteins have been extensively explored in this Special Issue. The technical developments of solid-state NMR, solution NMR, X-ray crystallography, florescence resonance energy transfer (FRET), linear dichroism (LD), and fluorescence correlation spectroscopy (FCS), etc., provide structural and functional studies of membrane proteins that are treated by detergents, micelles, liposomes, and nanodiscs, etc., as membrane mimic systems.

## Figures and Tables

**Figure 1:**
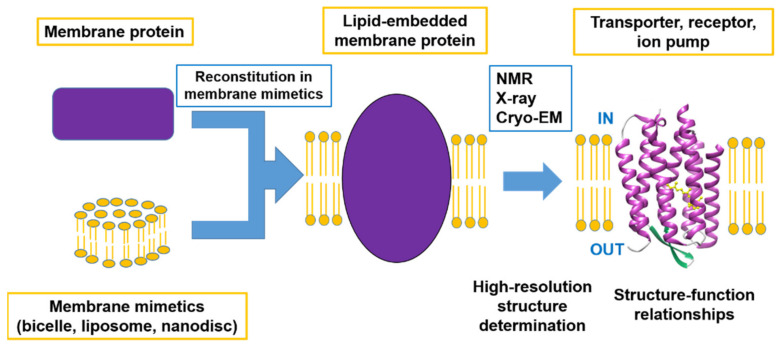
Schematic image of advanced research on the structure–function relationships of membrane proteins.
